# Rats and Mice

**Published:** 1916-02

**Authors:** 


### Rats and Mice.

In recent years attention has more and more been called to rats and mice as disease carriers. Some who have studied the question think they are far more dangerous than flies or mosquitoes. Certain it is that they are uncleanly and that they go from house to house tracking through all kinds of filth and leaving it as they go. Aside from their destructiveness, rats and mice should be exterminated because they are a menace to health.

No physician should ever allow himself to get too busy to read and study. No mind can improve without this necessary aid, and where there is no improvement there is degeneration.

Thanks are returned to the Charlotte (N. C.) *Medical Journal* for a much appreciated editorial notice of the Medical Package Library. Such kind words always encourage one to renewed efforts to deserve the nice things said.

Every physician should have some simple method of keeping books, a method that does not take too much time, but that shows exactly what the expenses and receipts are, and that will not allow a charge to be overlooked. Physicians are proverbially negligent about bookkeeping and also about collecting accounts.

---

And now comes Professor Lewis Wallin of the Psychological Clinic of St. Louis and says that according to the Binet-Simon "mind test," which has been in use for several years, most of us are feeble-minded, and that it is an abnormal adult that has a mind better than that of the average child of 13. That accounts, perhaps, for the apparent precocity of the present-day youth—he is smart only by comparison with the grown-ups.

---

The *Southern Clinic* of Richmond, Va., is publishing a very successful system for the collecting of doctors' bill. It is known as Bryce's Collector, and it carries a guarantee to "make you better off financially than you have been for years," or to refund the price of $1.00 paid for it. It contains 300 bills, and is so simple that an inexperienced office girl can use it. Write the *Southern Clinic* for sample pages. Remember the book will only cost $1.00 and you get a year's subscription to the *Southern Clinic* along with it.

---

The TEXAS MEDICAL JOURNAL is anxious to reach every physician in the State. It has most of the leading physicians on its subscription list, and would have many more if only those who read it and appreciate it would be thoughtful enough to recommend it to brother physicians, either privately or in public meetings. This would be but little trouble, and if the JOURNAL is really a good thing (as we believe it is) those who take this trouble would thus confer a double favor—on the one to whom they mention it and on the owner of the JOURNAL. Will you not do this much?

